# Comparative Antioxidant Efficacy of Green-Synthesised Selenium Nanoparticles From Pongamia pinnata, Citrus sinensis, and Acacia auriculiformis: An In Vitro Analysis

**DOI:** 10.7759/cureus.58439

**Published:** 2024-04-17

**Authors:** Archana Behera, Mukesh Kumar Dharmalingam Jothinathan, Iadalin Ryntathiang, Saantosh Saravanan, Ramadurai Murugan

**Affiliations:** 1 Centre for Global Health Research, Saveetha Medical College and Hospitals, Saveetha Institute of Medical and Technical Sciences, Chennai, IND

**Keywords:** antioxidant activity, phytochemical analysis, selenium nanoparticles, green synthesis, nanotechnology

## Abstract

Aim

This study aims to synthesise selenium nanoparticles (SeNPs) using extracts from *Citrus sinensis* peel (CSP), *Millettia pinnata* Leaf (MPL), and *Acacia auriculiformis* bark (AAB) as eco-friendly reducing agents. It seeks to compare the effectiveness of these plant extracts in the production of SeNPs and evaluate the antioxidant activities of the synthesised nanoparticles, establishing a link between the phytochemical constituents of the extracts and the antioxidant capacity of SeNPs for their potential applications in drug development and environmental sustainability.

Introduction

Nanotechnology offers innovative solutions in various fields, including medicine, environmental science, and materials engineering. SeNPs are of particular interest due to their unique properties and potential applications. The methods for synthesizing nanoparticles often involve hazardous chemicals, posing risks to the environment and human health. In response, green synthesis methods utilizing plant extracts have emerged as a sustainable alternative. This study focuses on utilizing CSP, MPL, and AAB extracts, rich in natural reducing agents such as flavonoids and phenolic acids, for the eco-friendly synthesis of SeNPs. These plant sources are chosen based on their known phytochemical profiles and potential antioxidant activities, and we aim to explore the correlation between the extracts' phytochemical composition and the antioxidant capabilities of the synthesised SeNPs.

Methods

SeNPs were synthesised using aqueous extracts of CSP, MPL, and AAB through a reduction process, in which selenium ions (Se^4+^) are reduced to elemental selenium. The presence of SeNPs was first visually monitored by colour change and then confirmed through UV-Vis spectroscopy and Fourier transform infrared (FTIR) spectroscopy analyses. The antioxidant activity of the synthesised SeNPs was assessed using the 1,1-diphenyl-2-picryl hydroxyl (DPPH) radical scavenging assay and the efficacy of SeNPs synthesised from different plant extracts was compared.

Results

The UV-Vis spectral analysis indicated a successful synthesis of SeNPs, as evidenced by the characteristic absorption peaks. The FTIR analysis confirmed the presence of organic molecules derived from the plant components on the outer layer of SeNPs, suggesting successful capping and stabilization of nanoparticles by phytochemicals in the extracts. Among the three types of SeNPs, those synthesised using *Citrus sinensis* peel extract (CSPE) exhibited the highest DPPH radical scavenging activity, indicating superior antioxidant properties compared to SeNPs synthesised from *Millettia pinnata* leaf extract (MPLE) and *Acacia auriculiformis* bark extract (AABE). This suggests that the antioxidant capacity of SeNPs is significantly influenced by the phytochemical composition of the plant extract used for synthesis.

Conclusion

The study highlights the potential of CSPE as an effective natural source for synthesising antioxidant-rich SeNPs and underscores the importance of green synthesis approaches in producing environmentally friendly and biologically active nanomaterials.

## Introduction

The crucial trace element selenium is necessary for numerous physiological processes. Selenium is an essential component of human diet [1]. Working with biological sources, such as plants, as a reducing agent, sodium selenite (Na_2_SeO_3_) is transformed into selenium nanoparticles (SeNPs) [2]. The nanoparticle synthesis using the green synthesis route, employing extracts from therapeutic plants, has gained particular significance recently. Plants are made primarily of phytochemicals, which readily form less harmful nanoparticles [3]. SeNPs are attracting attention for their unique properties and potential biological applications. While their production is well-documented, there is limited information on how reaction conditions and purification methods impact their physical characteristics, like size and shape, and the effectiveness of their antioxidant activity [4]. Fabaceae is a prominent and extensive group of angiosperms, comprising over 200 species. *Pongamia pinnata *or *Millettia pinnata* (*M. pinnata*) is a plant species belonging to the Fabaceae family, with a large distribution in India. Various components of this plant are utilised in medical practice to treat different disorders [5]. The advantage of utilizing affordable, natural, and recyclable materials is that metal oxide nanoparticles can be synthesised from *M. pinnata* extract without the use of hazardous chemicals or solvents [6].

SeNPs have antioxidant properties that can help protect cells and tissues from oxidative damage. Additionally, they prevent cellular damage caused by free radicals through their integration into antioxidant enzymes. Earlier studies have demonstrated that the leaf extract of *M. pinnata* is used as a reduction agent in the production of metal nanoparticles [7]. The ability of *M. pinnata-*synthesised metal oxide to scavenge free radicals was assessed using a variety of assays, including the 1,1-diphenyl-2-picryl hydroxyl (DPPH) assay [8]. Many species of *Millettia* produced a wide range of phytonutrients that have been identified as their secondary metabolites, including phenolic compounds, resins, polysaccharides, phytosterols, flavonoids, saponins, alkaloids, and terpenoids [9].

Citrus plants, belonging to the family Rutaceae, contain phenolic acids, flavonoids, organic acids, and dietary fibre [10]. A simple green route synthesis of SeNPs using the peel extract of *Citrus sinensis *(*C. sinensis)* is presented in this article. Indeed, metal nanoparticles synthesised using *C. sinensis* (orange) peel extract might possess medicinal properties [11]. *C. sinensis *peel has been proven in several studies to have antioxidant properties that may be used to enhance human health. The peel contains a high amount of bioactive chemicals, including ascorbic acid, flavonoids, carotenoids, phenolic compounds, and citric acid [12]. Peel oranges have a high level of antioxidant activity, which makes them a potential natural antioxidant food supplement or additive.

The *Acacia* genus has several species, which are widely distributed throughout the world. One of the Indonesian *Acacia* *species* is *Acacia auriculiformis (A. auriculiformi*s). The antioxidant qualities of *A. auriculiformis *bark have been highlighted in several studies [13]. The bark extract of *A. auriculiformis* showed good antioxidant potential in the DPPH and ferric-reducing antioxidant power (FRAP) assays [14]. Phytochemicals, including steroids, triterpenoids, tannins, flavonoids, alkaloids, glycosides, saponins, carbohydrates, and phenolic compounds, are present in the bark extract of *A. auriculiformis *[15].

To examine the antioxidant capacity of *C. sinensis *peel (CSP), *M. pinnata *leaf (MPL), and *A. auriculiformis *bark (AAB), an important and comparative evaluation based on their phytochemistry and biological activity has been carried out and reported in this study.

## Materials and methods

Collection of plant sources

The leaves of *M. pinnata *and one species of *Pongamia* were collected in Chennai, India, *C. sinensis *was collected from a local vendor in Chennai, and *A. auriculiformis *bark was collected from Balasore, Orissa, India. All the samples were collected between December 2023 and mid-January 2024. The samples were authenticated by the Centre for Advanced Studies in Botany at the University of Madras, Chennai, India.

Preparation of extracts

MPL, CSP, and AAB were chopped into small bits, rinsed two to three times with purified water, and kept in the shade for drying for three days. After they were completely dry, they were made into a powder and stored for further work. Then, 10 g of MPL, 5 g of CSP, and 5 g of AAB powder were each added separately into a 100 mL double-distilled H_2_O at 60°C for 20 min. Subsequently, the solution was filtered using muslin cloth followed by Whatman filter paper with a diameter of 125 mm. The filtered solution was then stored at a temperature of 4°C for subsequent processing.

Preparation of s​​​​odium selenite solution

The standard stock solution of Na_2_SeO_3_ (5mM) was prepared by dissolving it in 300 mL of distilled H_2_O.

Synthesis of selenium nanoparticles

**Figure 1:**
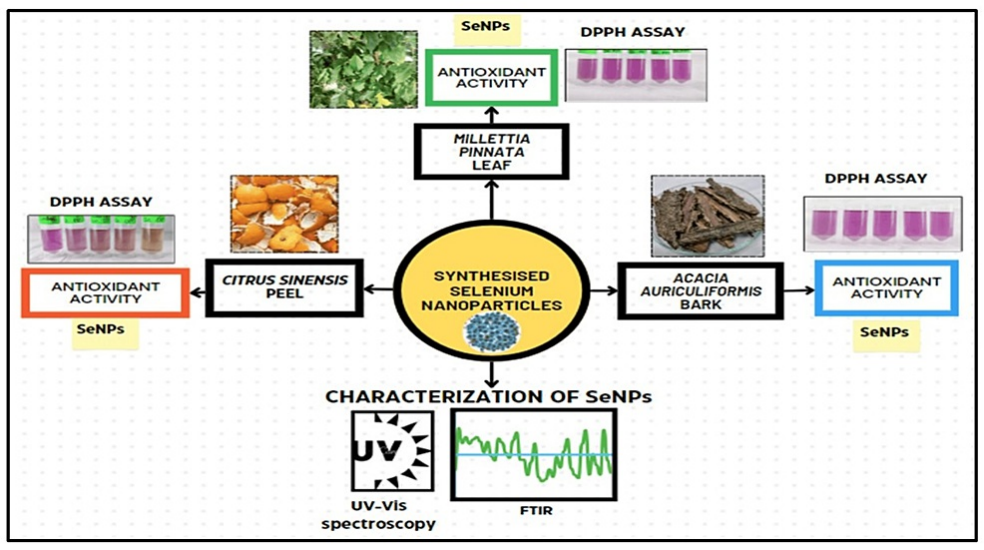
Green synthesis of selenium nanoparticles (SeNPs) by using plant extracts: Citrus sinensis peel, Millettia pinnata leaf, and Acacia auriculiformis bark Plant extracts: *Citrus sinensis *peel, *Millettia pinnata *leaf, and *Acacia auriculiformis *bark *Image credits: *Archana Behera SeNPs: selenium nanoparticles; DPPH assay: 1,1-diphenyl-2-picryl hydroxyl assay; FTIR analysis: Fourier transform infrared analysis

For SeNPs synthesis, about 10 mL (10%) of *M. pinnata *leaf aqueous extract (MPLE), *C. sinensis *peels extract (CSPE), and *A. auriculiformis *bark extract (AABE) were added separately to a 90 mL solution of Na_2_SeO_3_ (5 mM) and stirred at 35°C. The acidity level of the reaction mixture was continuously observed and documented. The reaction mixtures were placed in a dark environment on a rotating shaker at a temperature of 40°C and a speed of 300 rpm for 3 h. The colour of the reaction mixture was visually examined. The mixture underwent incubation at ambient temperature for 72 h. Following incubation, the colour of the reaction mixture underwent a complete transformation. The alteration in colour signifies the production of nanoparticles. The control remained unaffected with the addition of Na_2_SeO_3_, exhibiting no difference in colour. Figure 1 represents the process of synthesising SeNPs using three distinct plant extracts such as *M. pinnata* leaf, *A. auriculiformis* bark, and *C. sinensis* peel, along with the subsequent analysis of their properties and potential uses.

Characterisation of SeNPs

MPLE, CSPE, and AABE were used to synthesise stable SeNPs, while UV-visible spectroscopy and Fourier transform infrared (FTIR) spectroscopy were used to characterise the material. The absorption spectra of the reaction solution containing the synthesised SeNPs were studied by UV-vis spectrophotometry. The reduction of SeNPs was recorded under UV-visible spectroscopy using Labman Scientific UV-visible spectrophotometer (Labman Scientific Instruments Pvt. Ltd., Chennai, India) within the characteristic peak between 200 and 400 nm. The UV-visible spectra of MPLE, CSPE, AABE, and Na_2_SeO_3_ solutions were recorded. The functional properties of the nanoparticles were evaluated by FTIR analysis, which involved measuring the sample's light absorption at certain wavelengths ranging from 4000 to 400 cm^−1^. The various functional groups found in the samples were documented.

Antioxidant activity

DPPH Assay

The antioxidant activity of SeNPs synthesised by MPLE, CSPE, and AABE was examined using the DPPH free radical scavenging method using ascorbic acid as the standard. The DPPH radical-scavenging method is a straightforward technique that involves decolourising the solution. Upon the introduction of the oxidised form of DPPH ethanolic solutions (characterised by a deep violet colour) to the antioxidant molecule, a chemical reaction occurs, resulting in the reduction of DPPH and a subsequent colour shift from deep violet to yellow. A 2 mL of a standard solution of DPPH (0.1 mM) was added to 1 mL of SeNPs solution, which had been diluted with ethanol to achieve different concentrations (ranging from 30 to 60 μg/mL). Subsequently, the mixture was stirred and kept in a dark environment at ambient temperature. Following a 1 h incubation period, the radical scavenging activity was assessed by measuring the absorbance (A) of each solution at 517 nm using a UV-Visible spectrophotometer. The percentage inhibition (I (%)) was then calculated using the following equation:

$Percentage \hspace{00.2cm} of \hspace{00.2cm} inhibition= \left ( Absorbance \hspace{00.2cm} of \hspace{00.2cm}control \right )- \left ( Absorbance \hspace{00.2cm} of \hspace{00.2cm}sample \right ) \setminus \left (Absorbance \hspace{00.2cm} of \hspace{00.2cm}control \right )\times 100$

The measurement of the absorbance of both the control (A0) and the extractives/standard (A1) was documented. The percentage of inhibition was plotted against the concentration, and the IC_50_ value was determined from the graph. The method was replicated thrice at each concentration to ensure the precision of measurements [16].

Phytochemical test

Saponin Test

Foam test: A volume of 2 mL of the extract (MPL, AAB, and CSP) was dissolved in distilled water (2 mL) and vigorously mixed in a graduated cylinder for 15 min. Foam development is indicative of the presence of saponins.

Phenol Test

Ferric chloride test: A volume of 2 mL of the extract (MPL, AAB, and CSP) is treated with three to four drops of 6% ferric chloride solution. The formation of deep blue, black, and green colours indicates the presence of phenols and tannins.

Flavonoids Test

Alkaline reagent test: To 2 mL (MPL, AAB, and CSP) of the extract, a small amount of sodium hydroxide solution is added. The development of a vibrant yellow colour, which turns colourless upon the introduction of dilute acid, signifies the existence of flavonoids.

Glycosides Test

Legals test: A volume of 2 mL of the extract (MPL, AAB, and CSP) is treated with 2 mL of pyridine containing 1 mL of sodium nitroprusside solution. A small amount of NaOH solution is also added. The change in colour from pink to red signifies the existence of glycosides.

Steroids Test

Salkowski’s test: A volume of 2 mL of the extract (MPL, AAB, and CSP) is added to 5 mL of chloroform, followed by a few drops of concentrated H_2_SO_4_. A red-coloured low chloroform layer confirms the presence of steroids.

Amino Acid/Protein Test

Ninhydrin test: A volume of 2 mL of the extract (MPL, AAB, and CSP) and 2 mL of 0.25% ninhydrin reagent were added and kept in a water bath for a few minutes. The formation of blue colour indicates the presence of amino acids. The formation of a yellow colour indicates the presence of proteins. A deep blue, violet-pink, or red colour confirms the presence of amino acids.

Terpenoid Test

Bontrager’s test: A volume of 2 mL of the extract (MPL, AAB, and CSP) and 2 mL of chloroform were added and evaporated to dryness, followed by the addition of a few drops of concentrated H_2_SO_4_ and heated for 2 min. A red or reddish-brown colour confirms the presence of terpenoids.

Anthraquinone Glycoside Test

Nitric acid test: A volume of 2 mL of the extract (MPL, AAB, and CSP) and a few mL of nitric acid were carefully added to the walls of the test tube and kept in a water bath for 10-15 min. The colour change from yellow to orange confirms the presence of anthraquinone glycoside [17,18].

## Results

**Figure 2:**
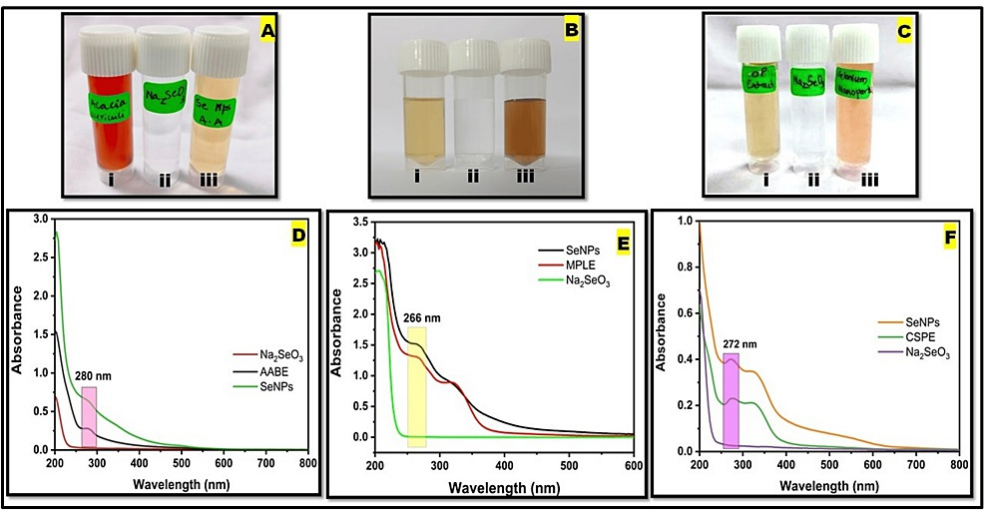
(A-C) shows the visible colour changes in green-synthesised selenium nanoparticles (SeNPs) by plant extracts: A(i) Acacia auriculiformis bark extract (AABE), A(ii) Na2SeO3 solution, A(iii) synthesised SeNPs; B(i) Millettia pinnata leaf extract (MPLE), B(ii) Na2SeO3 solution, B(iii) synthesised SeNPs; and C(i) Citrus sinensis peels extract (CSPE), C(ii) Na2SeO3 solution, C(iii) synthesised SeNPs, (D-F) show the UV-visible absorption spectrum of the green synthesis of SeNPs by plant extracts: (D) AABE, (E) MPLE, and (F) CSPE.

The nanoparticles underwent a colour transformation from cherry-red to pale yellow (Figure 2A(iii)), suggesting that AABE was responsible for the production of SeNPs. The NP solution underwent a colour transformation from whitish yellow to brownish orange (Figure 2B(iii)) due to the bioreduction of Na_2_SeO_3 _to SeNPs by MPLE. The aqueous extract derived from CSPE was utilised to perform the bioreduction of Na_2_SeO_3_ into SeNPs, leading to a noticeable alteration in colour from whitish yellow to yellowish orange in the solution containing the nanoparticles (Figure 2C(iii)).

The colour change was observed for all treatments with an increase in incubation times, but no additional colour changes were observed after 24 h of incubation at room temperature. Na_2_SeO_3_ solution (5mM) (vial "ii" in Figure 2A-2C) was kept as control without adding the plant extracts. The plant extracts are shown in vial "i" of Figure 2A-2C.

UV spectroscopy

After 24 h, the SeNPs synthesised by all the plant extracts showed absorption at 200-400 nm in UV spectroscopy. The SeNPs synthesised by AABE absorbed at 280 nm (Figure 2D), those synthesised by MPLE absorbed at 266 nm (Figure 2E), and those synthesised by CSPE at 272 nm (Figure 2F).

FTIR

**Figure 3:**
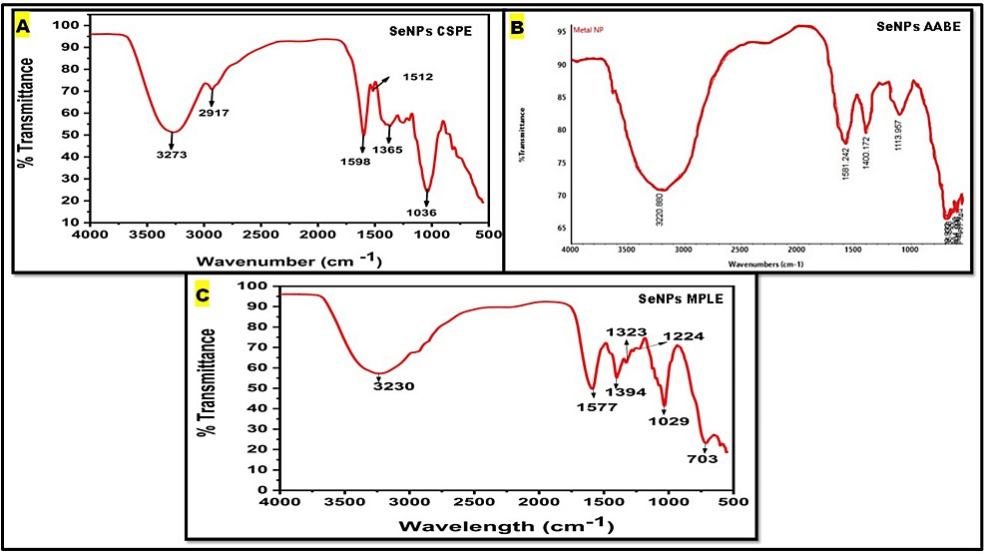
FTIR spectra of selenium nanoparticles (SeNPs) synthesised by plant extracts: (A) Citrus sinensis peels extract (CSPE), (B)​​​​​​​ Acacia auriculiformis bark extract (AABE), and (C)​​​​​​​ Millettia pinnata leaf extract (MPLE).

The individual biomolecules responsible for reducing, capping, and maintaining the stability of SeNPs in aqueous extracts were identified using FTIR analysis. The FTIR spectra were obtained within the wavenumber range of 400 to 4000 cm^−1^, as shown in Figures 3A-3C. The FTIR spectra of SeNPs synthesised by CSPE displayed stretching and vibrations at certain wave numbers: 3273, 2917, 1598, 1512, 1365, and 1036 cm^−1^ (Figure 3A). The peaks represent several functional groups such as N-H, O-H, N-O, C-H, C=C, S=O, and C-N, demonstrating the involvement of biomolecules in the synthesis and transformation of SeNPs. The presence of functional groups on SeNPs suggests that amines, alcohols, alkanes, phenol, sulfonate, and carboxylic acid serve as both reducing and stabilising agents for SeNPs. The SeNPs synthesised by AABE exhibit stretching and bending, with maxima shown at 3220, 1581, 1400, and 1113 cm^−1 ^(Figure 3B). The peaks represent several functional groups such as sulfonyl chloride, alcohol, carboxylic acid, sulfate, cyclic alkene, aliphatic ether, and amine. These functional groups suggest the involvement of biomolecules in the reduction and production of SeNPs. The SeNPs synthesised by MPLE (Figure 3C) exhibit peaks at 3230, 1577, 1394, 1323, 1224, 1029, and 703 cm^-1^, corresponding to various functional groups such as C=C, S=O, O-H, C-F, C-H, C-N, and C-Cl. These functional groups include alkane, carboxylic acid, sulfate, sulfonyl chloride, and phenol. Most of the functional groups used in the synthesis of SeNPs are identical.

Antioxidant activity

DPPH Radical Scavenging Assay

**Figure 4:**
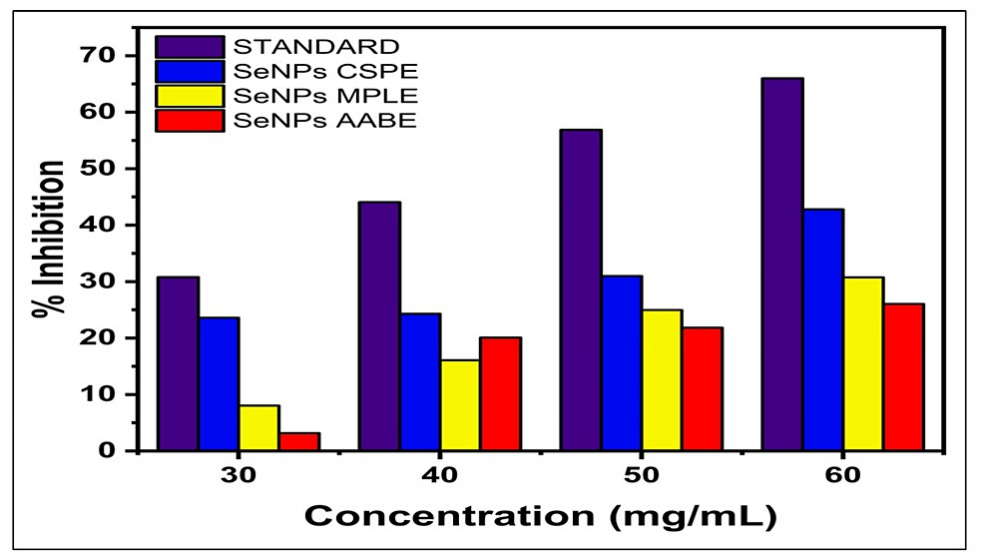
Antioxidant assay using DPPH of SeNPs synthesised by plant extracts: Millettia pinnata leaf extract (MPLE), Citrus sinensis peels extract (CSPE), and Acacia auriculiformis bark extract (AABE). Standard: Ascorbic acid SeNPs: selenium nanoparticles; DPPH assay: 1,1-diphenyl-2-picryl hydroxyl assay

The SeNPs synthesised by the aqueous extract of AABE, MPLE, and CSPE were evaluated using a DPPH free radical scavenging assay using ascorbic acid as the standard for investigating their antioxidant activity. Figure 4 demonstrates variations in the antioxidant capacity among the three SeNPs. The solutions directly interacted and decreased the diverse array of unpaired electrons of DPPH. The scavenging rate exhibited a positive correlation with the concentration of the tested sample, ranging from 30 to 60 µg/mL, in the presence of SeNPs. Based on our research, SeNPs synthesised using AABE, MPLE, and CSPE displayed high antioxidant effects. The IC_50_ value of CSPE-SeNPs was 8.49 μg/mL, which is a little higher than that of MPLE-SeNPs and AABE-SeNPs at 7.7 μg/mL and 7.1 μg/mL, respectively. The implication is that to some extent these activities may be dependent on the distinct chemical components of the extracts. These results highlight the importance of researching the synthesis of SeNPs and their interactions with specific phytochemicals to enhance the efficacy of plant-based antioxidants.

Phytochemicals test

**Figure 5:**
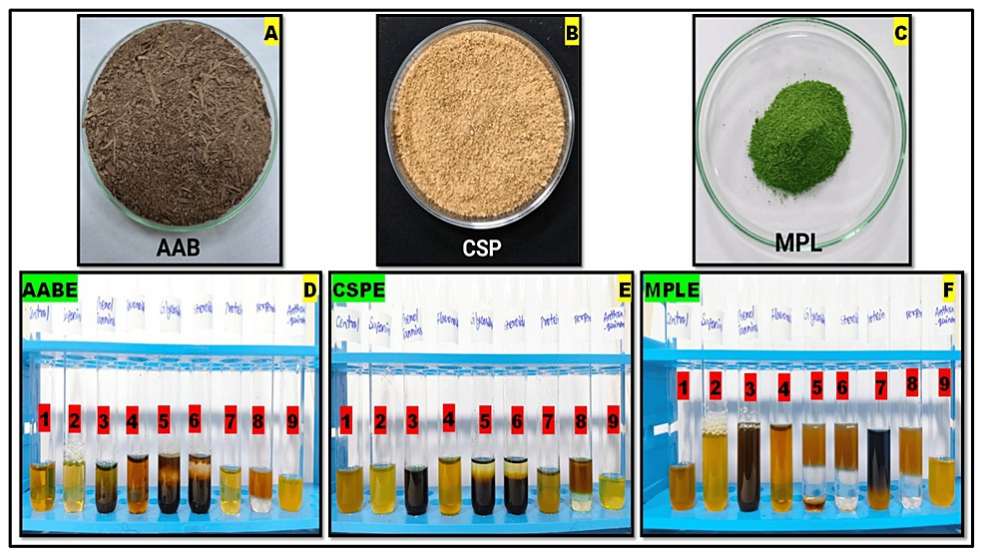
Phytochemical analysis of plant aqueous extracts (A) *Acacia auriculiformis *bark (AAB) powder, (B) *Citrus sinensis *peel (CSP) powder, (C) *Millettia pinnata *leaf (MPL) powder, (D) *Acacia auriculiformis *bark extract (AABE), (E) *Citrus sinensis *peel extract (CSPE), and (F) *Millettia pinnata *leaf extract (MPLE). (1) Aqueous extract control, (2) saponin, (3) phenol, (4) flavonoid, (5) glycosides, (6) steroid, (7) amino acid, (8) terpenoid, and (9) anthraquinone glycoside.

The dried powder of AAB, MPL, and CSP was collected (Figure 5A-5C) and the aqueous extract was stored at 4°C for further analysis. The phytochemical screening of all three extracts revealed the presence of prominent phytochemicals. Figure 5 displays the results. The chemical test indicated the presence of saponins, phenols, flavonoids, steroids, terpenoids, tannins, glycosides, and anthraquinones. Phytochemicals were found in AABE [15], MPLE [5], and CSPE [10], with similar outcomes. In the figure, the results of the phytochemical test are displayed as (1) the aqueous extract as control, (2) saponins, (3) phenol, (4) flavonoid, (5) glycosides (6) steroid, (7) amino acid, (8) terpenoid, and (9) anthraquinone glycoside. Figure 5D (1, 2, 3, 4, 5, 6, 7, 8, and 9) shows that AABE has phytochemical compounds such as saponins, phenols, tannins, protein, and glycosides along with control. Then CSPE in Figure 5E (1, 2, 3, 4, 5, 6, 7, 8, and 9) shows that this extract has phytochemical compounds such as phenols, tannins, glycosides, and terpenoids. The MPLE in Figure 5F (1, 2, 3, 4, 5, 6, 7, 8, and 9) shows that this plant extract has the phytochemical compounds saponins, phenols, tannins, steroids, and amino acids.

## Discussion

This study involved a comparison of the environmentally friendly production of SeNPs utilising a green synthesis method, with a focus on their antioxidant characteristics. We primarily focused on the prevention and treatment of several human diseases, including cancer, cardiovascular disease, inflammation, and other disorders caused by oxidative stress resulting from free radicals. Our research explored the potential of antioxidant therapy as a means to address these conditions in the coming years. We successfully synthesised SeNPs by adding Na_2_SeO_3_ solution to the aqueous extracts of AAB, CSP, and MPL separately [19, 20]. The characterisation techniques included UV-Vis spectroscopy and FTIR [21]. The study of SeNP synthesis was conducted using UV spectrophotometry and the results were recorded. The absorbance peak values were observed at 266 nm (MPLE), 280 nm (AABE), and 272 nm (CSPE), respectively.

The FTIR spectrum of SeNPs-CSPE showed peaks at 3273, 2917, 1598, 1512, 1365, and 1036 cm^−1^. The ranges of the absorbance indicated the presence of stretching N-H amide bonds, O-H bond alcohols, phenols, C=C, C-H, N-O, S=O, and C-N bonds. The FTIR spectrum of SeNPs-AABE displayed peaks at 3220, 1581, 1400, and 1113 cm^−1^ corresponding to C-O, C-F, S=O, O-H, C=C, and N-H bonds with carboxylic acid, amine, alcohol, sulfate, etc. The FTIR spectrum of SeNPs-MPLE exhibited peaks at 3230, 1577, 1394, 1323, 1224, 1029, and 703 cm^-1^, corresponding to various functional groups such as C-H, S=O, C=C, O-H, C-F, C-N, and C-Cl with alkane, carboxylic acid, sulfate, sulfonyl chloride, and phenol. The aqueous extracts of AAB, CSP, and MPL contained essential phytochemicals such as steroids, tannins, amino acids, flavonoids, saponins, proteins, and phenols. In our study, CSPE has a higher number of phytochemicals than AABE and MPLE. These functional groups played a crucial role in capping and bioreduction, contributing to nanoparticle stability. The results exhibit a strong correlation with the findings of earlier studies [5,10,15].

When we compared the properties of SeNPs synthesised from all three extracts, SeNPs synthesised by CSPE showed the highest antioxidant activity. The evaluation of the antioxidant potential of SeNPs was done using the DPPH assay. Their ability to scavenge free radicals highlights their potential as natural antioxidants [4,21]. A similar study by Nagalingam et al. [22] reported that SeNPs exhibited significant dose-dependent free radical inhibition, achieving 66.7% inhibition at a concentration of 10 μL, which increased to 83.7% at a concentration of 50 μL. Chellapa et al. [23] found that SeNPs demonstrated superior antioxidant capabilities compared to ascorbic acid at all tested doses. The effectiveness of SeNPs was directly proportional to the dosage, as a concentration of 50 µl/ml resulted in a 93.15% inhibition rate. Zhang et al. [24] demonstrated that SeNPs functionalised with 1% green tea *Lycium barbarum* polysaccharides (LBP-GT) significantly neutralised DPPH/2,2-azinobis(3-ethylbenzothiazoline-6-sulfonic acid) diammonium salt (ABTS) radicals, especially at 5-25 μM, surpassing Na_2_SeO_3 _and LBP with up to 52.5% DPPH scavenging, proving to be efficient than green tea extracts.

Limitations

Despite the promising antioxidant potential of SeNPs, limitations include the variability in the synthesis and antioxidant activity depending on the plant extract used, potential cytotoxicity at higher concentrations, and the need for further in vivo studies to confirm efficacy and safety in humans.

## Conclusions

This study aimed to compare and analyse the antioxidant capabilities of SeNPs that were synthesised using different plant extracts. Notably, SeNPs synthesised using CSPE exhibited the highest antioxidant activity. While both SeNPs synthesised by AABE and MPLE showed antioxidant potential, they were outperformed by the CSP-synthesised SeNPs. These findings highlight the potential of natural sources, specifically citrus peel, for the green synthesis of SeNPs with remarkable free radical scavenging capabilities. Further exploration of the reducing, capping, and stabilising abilities of these SeNPs could provide valuable insights into their role in cellular metabolism and potential applications.
